# Unleashing the link between the relaxation of the COVID-19 control policy and residents’ mental health in China: the mediating role of family tourism consumption

**DOI:** 10.3389/fpubh.2023.1216980

**Published:** 2023-08-22

**Authors:** Yilun He, Shaowen Zhan, Hui Su, Yulong Deng

**Affiliations:** ^1^School of Management, Xi’an University of Architecture and Technology, Xi’an, China; ^2^School of Public Administration, Xi'an University of Architecture and Technology, Xi'an, China

**Keywords:** relaxation of COVID-19 control, pressure, anxiety, depression, tourism consumption, China

## Abstract

**Objective:**

COVID-19 has negatively influenced industrial development, family consumption, and residents’ mental health. Unfortunately, it has not yet been studied whether this adverse situation can be alleviated after the relaxation of the COVID-19 control policy (RCC). Therefore, this study aimed to analyze the effect of the RCC on the resident’s mental health and the mediating effect of family tourism consumption.

**Methods:**

By using the PSM and mediating effetc model to research the panel data of two periods (April 2021 and April 2023) for Shaanxi province, China.

**Results:**

The RCC negatively inhibited the mental health severity of residents, and the mental health severity decreased by 0.602. In particular, the RCC showed the most substantial negative effect on residents’ stress, followed by anxiety and depression. Meanwhile, it is found that the impact of the RCC on the mental health of residents is highly heterogeneous. The RCC indicates a linear significant effect on the mental health of residents under 60 years of age, while the results were found insignificant for residents above 60 years of age. Meanwhile, the RCC’s improvement effect on urban residents’ mental health is greater than that of rural residents. In addition, mechanism analysis showed that tourism consumption plays a mediating role in the influence of the RCC on the mental health of residents, and the mediating effect accounted for 24.58% of the total effect.

**Conclusion:**

Based on the findings, the study proposes that government and policymakers should strengthen mental health intervention, improve access to mental health counseling, stimulate economic development, expand the employment of residents, and track the mutation of the novel coronavirus.

## 1. Introduction

The ravages of COVID-19 are reflected in the increasing number of daily deaths and infections and in the distortion of residents’ mental health, which has become a global public health issue that needs to be intervened in the post-COVID-19 era (1, 2). Previous studies have confirmed the negative influence of COVID-19 on residents’ mental health; for instance, Chen et al. (3) surveyed 18,171 people from 35 countries/societies and found that about 26.6% of the residents had moderate to extreme depressive symptoms, 28.2% of the residents had moderate to severe anxiety symptoms, and 18.3% of the residents had moderate to extreme stress symptoms due to the spread of COVID-19. Later, a follow-up study of 1,161 Americans found that the prevalence of severe depressive symptoms increased from 27.8% in March 2020 to 32.8% in April 2021, and the increase was even more significant among low-income groups (4). Moreover, from the end of January to the middle of April 2020, the detection rates of anxiety and depression in Chinese samples were 29.6 and 32.5%, respectively, both significantly higher than the levels of pre-epidemic epidemiological surveys (5). Similarly, other studies [see Rufus et al. (6), Ramos et al. (7), and Dyer et al. (8)] found that the COVID-19 epidemic has significantly increased the rate of mental health disorders, especially among residents in areas with poor economic conditions. Besides, other studies also analyzed the impact of COVID-19 on the mental health of special groups, such as doctors, students, older people, and low-income groups (9–11). It is also revealed that the mental health costs of COVID-19 are huge. The large number of mental health cases has strained medical resources, treatment costs, and financial burdens in the era of COVID-19 (12). Additionally, inefficient work or reduced productivity due to mental health problems created new groups of poor that inhibited the sustainable growth of GDP (13). Thus, it is found that COVID-19 and its ramifications have caused stress, anxiety, and depression worldwide. In the post-pandemic era, alleviating residents’ mental health is significant for coping with new global public health problems and boosting economic and social development.

Moreover, much of the literature explored why COVID-19 affects mental health. Firstly, COVID-19 control policies such as wearing masks, restricting travel, keeping social distance, and quarantine policies have changed the way residents live, which may directly impact the mental health of residents through difficulties in breathing fresh air, increase in lonely time, and decrease of human interaction, as well as fear of isolation policy (14–16). Secondly, residents, especially those with serious underlying diseases, are worried and afraid that the COVID-19 epidemic is highly contagious and has ambiguous sequelae. No vaccine can prevent 100% of people from getting infected (17–19). Thirdly, job insecurity or economic uncertainty due to COVID-19 lockdown and quarantine policies increases residents’ stress and anxiety (20, 21). Fourthly, residents’ fear is aggravated by the shortage of medical resources and the concern of cross-infection in the hospitals (22). Finally, public discrimination against people infected with COVID-19 in employment or social communication is also an essential reason for residents’ depression (23). Consequently, government and social organizations have taken targeted intervention measures, such as providing psychological services, supporting the development of the Internet economy, promoting flexible employment of residents, and timely disclosure of COVID-19 infection information (24–26). In addition, several studies have focused on exploring the phenomenon by taking samples of respondents such as doctors, workers in quarantine hotels & airports, and customs inspectors, who are more vulnerable to COVID-19 and are more likely to have serious mental health issues (16, 27–30). Therefore, although the influence mechanism of COVID-19 on residents’ mental health is more complex, there is a clear causal relationship between them. Further, will this causality break down as COVID-19 containment policies are lifted? Previous empirical studies have not given a clear answer. So, to answer more clearly, the current research will contribute innovatively to the existing body of knowledge.

It is further believed that the COVID-19 pandemic has dampened the tourism industry and family tourism consumption due to travel restrictions, quarantine policies for suspected infections, and social distancing. The crash in international tourism due to the coronavirus pandemic also caused a considerable loss of more than $4 trillion to the global GDP for 2020 and 2021, according to a UNCTAD report published on 30 June (30). Likewise, the study of Martin et al. (31), using the dynamic CGE modeling framework, also revealed a sharp decline in number of tourists for the year 2020. The study showed that the value of tourism in Tanzania decreased by more than 13%, and total labor demand for tourism and related industries also declined by more than 3.3 percent in 2021. Moreover, COVID-19 has prompted residents to adopt a cautious travel attitude, reduce public transportation use, reduce travel time, lessen international tourism, and prefer outdoor or short-distance travel (32, 33). Accordingly, the COVID-19 pandemic has significantly reduced the number of tourists and the output value of the tourism industry and related industrial chains. Moreover, it also significantly decreased the ability to absorb employment and severely impacted the development of the tertiary industry and the job market in many countries, especially those that rely on tourism income (34–36). Of course, COVID-19 has also accelerated the adjustment of the tourism industry and the market competition pattern, such as digital tourism, innovation in tourism products, and the improvement of tourism services. These changes also provide a good opportunity to recover the tourism industry and consumption after the COVID-19 epidemic (37).

Although the tourism industry is a sunrise industry driven by the spiritual pursuit of residents, some scholars hold that long-distance travel requires a variety of individuals skills, such as physical fitness, concentration, understanding, decision-making, and confidence, and that mental health can affect the acquisition of these skills, which can affect tourists’ sense of experience, satisfaction, and happiness (38–40). However, other studies have confirmed the unique role of residents’ travel consumption in relieving stress, anxiety, or depression (41–43). Tourism consumption can improve residents’ mental health by contacting the beautiful nature, accepting the improvement of culture, relaxing the body, and forgetting their troubles (44, 45). Similarly, Liu et al. (46) evaluated the mental health of 89 anxious and 72 depressed tourists and stated that tourism consumption significantly reduces the anxiety of tourists. Based on the literature analysis, Cheng et al. (47) found that tourism consumption can significantly reduce the sense of burnout and pressure of tourists and improve their sleep and mental health. In addition, Sun et al. (48) also argued that tourism consumption has a significant inhibitory effect on family members’ negative emotions or mental illness. Besides, some scholars hold a neutral attitude and argue that the causal relationship between tourism and mental health should be interpreted cautiously, as reverse causality may lead to endogenous issues (49). However, existing studies have not yet considered the role of tourism consumption in the influence of the RCC on residents’ mental health.

In summary, previous studies have not empirically tested the causal relationship between RRC and residents’ mental health. Moreover, the mediating mechanism of family tourism consumption has not been studied previously. To make up for the research gaps, the study’s main objects is to innovatively explore the effect of the RCC on residents’ mental health and the mediating effect of family tourism consumption. Furtherly, the main contribution include the following: First, we employed the differences-in-differences (DID) method to explore the impact of the RCC on residents’ mental health using micro-panel data from Shaanxi province, China. Second, considering age and urban–rural differences, this paper explored the heterogeneity of the effects of the RCC. Third, the mediating effect model was used to test the role of family tourism consumption concerning the impact of the RCC on residents’ mental health. Finally, the study provides valuable experience for other countries or governments to improve the mental health of residents in the post-epidemic era.

## 2. Literature review and hypotheses development

### 2.1. Conservation of resources (COR) theory

Psychologists generally hold that individuals would continuously pursue happiness and success. Individuals are more likely to succeed if they can establish and maintain the personal characteristics and social status that can lead them to higher incomes and protect them from losses (50). Further, Hobfoll (51) proposed the COR theory, which mainly described the role of resources in the interaction between individuals and the social environment. COR theory limits the concept of resources to material resources (such as family assets and property), conditional resources (such as marriage and power), personality traits (such as self-efficacy and self-esteem), and energy resources (such as time and labor). The core notion behind this theory is that individuals strive to acquire, maintain, and protect resources they deem valuable. Suppose these resources are at risk of loss because of a stressful event. In that case, individuals prefer to adopt appropriate strategies such as collective action, social support, and optimal allocation of resources to minimize the damage (52). Therefore, the conservation of resources, loss of resources, and the actions taken result from the individual’s stress response. In recent years, COR theory has been used to test stress response and individual coping strategies under the influence of crisis (53, 54). In the past 3 years, the COVID-19 epidemic and control policies have become the biggest external shocks that individuals face, which may directly reduce resource access opportunities, value-added, and resource allocation efficiency and exacerbates the severity of an individual’s mental health. Furthermore, if COVID-19 control policies are relaxed, residents’ mental health can improve. Besides, the formation of an individual’s mental health is not short-term but the result of the long-term action of underlying risk factors (55–57). Individuals would inevitably adopt appropriate family strategies such as family travel consumption to adjust family resource allocation and alleviate long-term mental health problems. Therefore, this paper incorporated the RCC, residents’ mental health, and family tourism consumption into the COR theoretical analysis framework.

### 2.2. RCC and residents’ mental health

According to COR theory, individuals experience emotional feedback when they perceive the threat of losing resources or experience the actual loss of resources (51, 58). Welfare economics holds that the most essential resources in the market are the welfare resources owned by individuals and families. Previous studies have described welfare resources mainly from the perspective of family economy, social security, social network, and psychological conditions (59). If COVID-19 control policies are relaxed, welfare resources can be protected, and increase in value, and mental health problems such as stress, anxiety, or depression caused by residents’ fear of resource loss will gradually be lessened; the RCC is suitable for raising residents’ family income. In the post-epidemic era, residents can obtain more job opportunities, and wage income growth can become an essential guarantee for improving residents’ mental health (60, 61). Meanwhile, compared to the pandemic, the market potential of household fixed assets preservation and appreciation was better (62). The RCC can help to enhance the social security of the residents. The stress, anxiety, and depression of residents are mainly due to concerns about social insecurity, such as the epidemic’s infection rate and death rate, as well as loneliness due to isolation control and social discrimination against infected people (14, 63, 64). Third, the RCC could enhance the relationship network of residents. Removing social distancing and isolation policies can lessen the density and intensity of residents’ relationship networks. Many studies have further confirmed that the relationship network is crucial for improving mental health concerns (65–67). The RCC has significantly enhanced the psychological conditions of residents. Good psychological conditions, such as being respected and confident about the future, are essential to improve residents’ mental health (68, 69). Besides, studies also found that the psychological resilience of older adults with underlying diseases is very weak, and it is difficult for them to get rid of anxiety and depression quickly (70, 71). Meanwhile, compared with rural areas, cities showed higher population density and frequent mobility, with a higher risk of COVID-19 infection. The epidemic affected residents’ economic status and mental health more (72, 73). Accordingly, the RCC affected urban residents more than those in rural areas. Therefore, we propose the following hypothesis.

*H1*: The RCC could significantly improve the mental health of residents

*H1a*: Compared to non-older adults, the RCC had a weak effect on improving mental health in the older adults.

*H1b*: Compared with rural residents, the RCC had a stronger effect on improving the mental health of urban residents.

### 2.3. The mediating effect of family tourism consumption concerning the impact of the RCC on residents’ mental health

COR theory holds that individuals are not passive in coping with resource loss and psychological stress but can adjust their strategies according to the resource situation (51). Family tourism consumption is an essential strategy to optimize the allocation of family resources by improving residents’ sense of experience, freedom, and happiness, which can relieve residents’ stress, anxiety, and depression caused by COVID-19 (74, 75). Specifically, first, the RCC enhanced the financial support for family tourism consumption by increasing employment opportunities, unblocking employment channels, and directly improving family income (76, 77). Second, the RCC abandoned control measures such as social distancing, home restrictions, and hotel isolation to ensure the time and space required for family travel, which became an essential policy condition for developing the tourism industry after the epidemic (78, 79). Finally, the RCC has stimulated residents’ pursuit of a better and more enjoyable life in the past 3 years. Tourism is known as a pastime, enjoyment, and relaxation activity, especially outdoor natural scenery tourism, which could help to improve long-term residents’ depressed psychology and negative emotions (80, 81). Besides, to avoid the causal debate between tourism and mental health, the tourism value theory holds that leisure is the primary function of residents’ tourism consumption (82). To get rid of the hustle and bustle of the city, busy work, and tedious family affairs, residents’ travel experience is to change their way of life and experience another kind of beauty in real life (83, 84). Therefore, we infer that the RCC affected residents’ mental health by stimulating family tourism consumption and proposes the following hypothesis.

*H2*: Family tourism consumption had an intermediary effect concerning the RCC's influence on residents' tourism consumption.

## 3. Materials and methods

### 3.1. Study sites, sampling, and participants

The study included the panel data for the two periods, April 1 to 7, 2021, and April 1 to 7, 2023, respectively, through a large-scale online survey of residents from Shaanxi Province, China. The main reasons for the selection of sample areas are as follows: Shaanxi Province is in the west of China, the income gap between urban and rural residents is large, and the differentiation of urban and rural sample areas is obvious. Moreover, the research group won the cooperation with China Mobile Shaanxi Co., LTD. to carry out online questionnaire survey. Specially, firstly, the Mobile Shaanxi Co., LTD. randomly informed residents of the purpose, main content, and rewards for phone calls through mobile phone messages. Secondly, if the respondent agreed, the interviewer contacted them by phone to complete the questionnaire. Finally, the research group protected the transferee’s mobile phone number information and collected panel data. The questionnaire’s main contents included the respondents’ characteristics, the characteristics of their families, their cognitive status, their employment and income, and mental health. Excluding 42 respondents who were unwilling to answer during the second survey, the questionnaire survey obtained data from 735 residents, among which 421 belonged to urban areas while 314 were from rural areas. Besides, since the questionnaire adopted the principle of random sampling and the mobile phone coverage rate of urban and rural residents in Shaanxi exceeded 90%, sample selection bias was minimized.

### 3.2. Variable selection

#### 3.2.1. Dependent variable

In this study, the dependent variable is the residents’ mental health. Previous literature has characterized the residents’ mental health mainly from stress, anxiety, and depression. Referring to relevant studies such as Zhou et al. (85) and Huang et al. (86) and the time-saving requirements of online questionnaire survey, we selected some representative indicators, such as quality of sleep (very poor =1—very good =5), feeling sad (very rare =1—very often =5), concentration at work (very poor =1—very good =5) to measure residents’ press; cranky (very rare =1—very often =5), tachycardia (very rare =1—very often =5), and fidget (very rare =1—very often =5) to characterize residents’ anxiety; hard to do anything (very rare =1—very often =5), with no hope for life (very rare =1—very often =5), and suicidal thoughts in head (very rare =1—very often =5) to depict residents’ depression. The severity of the mental health issue of the residents was obtained by averaging nine indicators. Cronbach ‘s alpha was 0.82, signifying these indicators has good reliability. Additionally, “sleep quality” and “concentration at work” were positive indicators, so we conducted a reverse-coding calculation to maintain the same standard measurement as other indicators. Table 1 provides a descriptive statistical analysis of the mental health of the residents.

**Table 1:** Descriptive statistical analysis of residents’ mental health.

Variables	Mean	Min	Max	S.D.
Stress				
Quality of sleep	4.152	1	5	0.706
Feeling sad	3.623	1	5	0.501
Concentration at work	3.845	1	5	0.512
Anxiety				
Cranky	3.062	1	5	0.403
Tachycardia	2.951	1	5	0.392
Fidget	3.753	1	5	0.593
Depression				
Hard to do anything	2.796	1	5	0.405
With no hope for life	2.655	1	5	0.402
Suicidal thoughts in head	2.032	1	5	0.396

#### 3.2.2. Independent variable

The independent variable is the relaxation of COVID-19 control policy, categorized by the dummy variable. In December 2022, China relaxed its prevention and control measures and lowered the epidemic from A to B. Meanwhile, the novel coronavirus pneumonia was renamed novel coronavirus infection. Therefore, we assigned a value 1 to samples collected before December 31, 2022, and 0 to samples collected after that date.

#### 3.2.3. Control variables

Referring to the studies of Xiong et al. (25) and Albikawi (64), the study also included control variables, such as sex, age, education level, risk preference, awareness of COVID-19, the proportion of the older adults and children, job satisfaction, disposable income *per capita*, time to pay attention to COVID-19, chronic diseases, relationship network, urban or rural areas. Additionally, considering that other policy factors might directly influence the residents’ mental health, the study added the influence of government mental health counseling on residents’ mental health. The study used independent sample T to test the difference between samples A and B, and the results are reported in Table 2.

**Table 2:** Descriptive statistics of all variables.

Variables	Variable assignment	Sample A (2021.04)	Sample B (2023.04)	Difference (B-A)
Mental health level	The mean value of 9 indicators in Table 1	3.648	3.037	−0.611**
Stress	The mean value of 3 indicators in Table 1	4.368	3.378	−0.990*
Anxiety	The mean value of 3 indicators in Table 1	3.901	3.419	−0.482**
Depression	The mean value of 3 indicators in Table 1	2.675	2.313	−0.362*
RCC	Dummy variable (samples obtained before December 31, 2022, are assigned a value of 0, otherwise 1)	0	1	1.000
Gender	Man = 1, woman = 0	0.652	0.652	0.000
Age	Actual age (year)	39.452	41.452	2.000
Education level	School time (year)	11.250	12.035	0.785
Risk preference	Risk aversion = 1, risk neutrality = 2, risk pursuit = 3	1.782	1.901	0.119
Awareness of COVID-19	Do you realize the harm of COVID-19 and the self-limiting nature of the virus? (Yes = 1, no = 0)	0.475	0.801	0.326**
Proportion of the older adults and children	The proportion of people over 60 years old and children under 6 years old in the total household population (0–1)	0.402	0.415	0.013
Job satisfaction	Very dissatisfied =1—very satisfied =5	2.135	3.896	1.761***
*Per capita* disposable income	Disposable income in the first quarter (2500–5,000 yuan =1, 5,001–7,500 yuan =2, 7,501–10,000 yuan =3, 10,001–12,500 yuan =4, more than 12,500 yuan =5)	1.905	1.946	0.041**
Time of paying attention to COVID-19	0–15 min =1, 16–30 min =2, 31–45 min =3, 46–60 min =4, longer than 60 min =5	3.015	1.272	−1.743*
Chronic diseases	Whether you have a chronic disease (Yes = 1, no = 0)	0.402	0.411	0.009
Relationship network	How many regular contacts do you have each month? (Less than 10 = 1, 11–20 = 2, 21–30 = 3, 31–40 = 4, more than 40 = 5)	1.755	2.369	0.614**
Urban or rural areas	Urban residents = 1, rural residents = 0	0.573	0.573	0.000
Mental health counseling	Have you received mental health counseling from the government? (yes = 1, no = 0)	0.721	0.522	−0.199*

*, **, and *** represent significance at the 10, 5, and 1% levels, respectively.

According to Table 2, it is found that there is a significant difference in residents’ mental health before and after the RCC. Overall, the mental health level of residents increased by 0.611. The differences in stress, anxiety, and depression between the different groups are-0.990, −0.482, and-0.362, respectively, indicating that the mental health of the residents improved significantly after RCC. In addition, some other control variables also differed considerably between the sample groups. Compared with residents under COVID-19 control, after the COVID-19 control was relaxed, residents had a more precise awareness of COVID-19 (*diff*. = 0.326**), and they could not only realize the harm of COVID-19 but also treat the self-limiting disease objectively and rationally. Residents’ job satisfaction is also higher (*diff*. = 1.761***), and *per capita* disposable income has increased significantly (*diff.* = 0.041**). Moreover, residents have a more robust network of relationships (*diff*. = 0.614**). Further, they have substantially less time to pay attention to COVID-19 (*diff.* = −1.743*) and are less likely to receive mental health counseling from the government (*diff.* = −0.199*).

### 3.3. Empirical methods

#### 3.3.1. Difference-in-difference method

Since the outbreak of COVID-19 in December 2019, the Chinese government has adopted the class A management strategy for class B infectious diseases and implemented various control measures such as maintaining social distancing, wearing masks, and shutting down the infected areas. Until December 2022, the 3-year epidemic has seriously affected the mental health of residents. As the Omicron virus became less virulent, the Chinese government has adjusted and relaxed COVID-19 control. An issue worth studying is whether the mental health of residents is restored after the relaxation of the COVID-19 control policy. What are the possible mediating mechanisms? To this end, first, using the DID model, two periods of panel data from residents of Shaanxi, China, were used to empirically analyze the influence of the RCC on residents’ health levels. The model is constructed as follows:


(1) $$Y_{it}=\alpha_{0}+\alpha_{1}RCC_{it}+\Sigma \gamma X_{it}+\mu_{i}+\nu_{i}+\varepsilon_{it}$$

Where $Y_{it}$ signifies residents’ mental health, including stress, anxiety, and depression. i and t meant county and year. RCCsignifies relaxation of COVID-19 control. $X_{it}$ indicates control variables, and Σγ, is the effect of control variables on residents’ mental health. $\alpha_{0}\;$ is the constant term and $\;\alpha_{1}$ is the effect of the RCC on residents’ mental health. $\mu_{i}$ and $\nu_{i}$ are locality and time-fixed effect, respectively. $\varepsilon_{it}\;$ signifies the random error term. In addition, since two-term panel data was used, and interviewee were the same individuals at different times. So, we fixed regional and temporal differences, but not individual heterogeneity.

#### 3.3.2. Mediating effect model

Based on model verification (1), tourism consumption is further added to empirically test that the RCC could significantly improve residents’ mental health by improving family tourism consumption. The mediating variable “tourism consumption” is selected mainly given the following considerations: after the deregulation of COVID-19 control, the tourism industry showed retaliatory recovery and development. Meanwhile, travel is also a core mean to improve residents’ mental health. Consequently, the study employed the mediating effect model to analyze the mediating effect of tourism consumption. The hierarchical regression is constructed as follows:


(2) $$\begin{array}{c} Y_{it}=\alpha_{0}+\alpha_{1}RCC_{it}+\Sigma \gamma X_{it}+\mu_{i}+\nu_{i}+\varepsilon_{it}\;\\ M_{it}=\varphi_{0}+\varphi_{1}RCC_{it}+\Sigma \gamma X_{it}+\mu_{i}+\nu_{i}+\varepsilon_{it}\;\\ Y_{it}=\eta_{0}+\eta_{1}RCC_{it}+\eta_{2}M_{it}+\Sigma \gamma X_{it}+\mu_{i}+\nu_{i}+\varepsilon_{it} \end{array}$$

Where M signifies mediating variable “tourism consumption, “$\varphi_{0}$ and $\eta_{0}$ are constant terms, and $\varphi_{1}$, $\eta_{1},$ and $\eta_{2}$ are coefficients to be estimated. The meanings of other variables are the same as in formula (1). The specific testing process of mediating effect is the same as Si et al. (87).

## 4. Results

### 4.1. Influence of the RCC on the mental health of residents

According to Table 3, it is apparent that the RCC could significantly improve residents’ mental health and reduce the severity of mental health issues by 0.602. In particular, the RCC holds a negative inhibitory effect on residents’ stress, anxiety, and depression, and the severity of stress, anxiety, and depression decreased by 0.805, 0.406, and 0.315, respectively. Hence, the RCC has shown the strongest negative effect on residents’ anxiety, followed by anxiety and depression. Thus, hypothesis H1 is confirmed.

**Table 3:** The effect of RCC on the mental health of residents.

Variables	Mental health	Stress	Anxiety	Depression
RCC	−0.602** (0.283)	−0.805*** (0.251)	−0.406* (0.214)	−0.315* (0.170)
Gender	−0.206* (0.114)	−0.192* (0.102)	−0.201* (0.114)	−0.179* (0.098)
Age	0.108 (0.077)	0.185 (0.142)	0.094 (0.058)	0.062 (0.041)
Education level	0.301 (0.188)	0.362 (0.249)	0.325 (0.232)	0.204 (0.127)
Risk preference	−0.008 (0.005)	−0.006 (0.004)	−0.007 (0.005)	−0.006 (0.005)
Awareness of COVID-19	−0.406** (0.176)	−0.504** (0.229)	−0.401** (0.174)	−0.312** (0.138)
Proportion of the older adults and children	0.291*** (0.097)	0.125*** (0.040)	0.306*** (0.109)	0.291 (0.223)
Job satisfaction	−0.505* (0.280)	−0.321* (0.178)	−0.622* (0.347)	−0.106 (0.070)
*Per capita* disposable income	−0.219** (0.099)	−0.435** (0.197)	−0.006 (0.004)	−0.012 (0.008)
Time of paying attention to COVID-19	0.038* (0.021)	0.005 (0.003)	0.012* (0.007)	0.023* (0.012)
Chronic diseases	0.072 (0.045)	0.056 (0.040)	0.039 (0.026)	0.064 (0.043)
Relationship network	−0.208** (0.090)	−0.312** (0.156)	−0.231** (0.110)	−0.257** (0.109)
Mental health counseling	−0.031* (0.018)	−0.102* (0.056)	−0.061 (0.038)	−0.057 (0.040)
Constant term	0.506*** (0.017)	0.492*** (0.164)	0.394*** (0.127)	0.515*** (0.160)
Fixed area (Urban or rural areas)	Yes	Yes	Yes	Yes
Fixed time (2021.04 or 2023.04)	Yes	Yes	Yes	Yes
R^2^	0.506	0.621	0.645	0.602

*, **, and *** represent significance at the 10, 5, and 1% levels, respectively. Robust standard error is reported in parentheses.

Additionally, it is believed that the mental health of the residents is also affected by certain other factors. For instance, the gender of the residents, the awareness of COVID-19, and job satisfaction are also likely to significantly and negatively influence the mental health of the residents. If the residents surveyed were men, their mental health severity would decrease by 0.206; If residents realized the harmfulness of COVID-19 and the self-limiting nature of the virus, their mental health severity would decrease by 0.406; If the residents’ job satisfaction increased by 1 unit, the severity of the residents’ mental health would decrease by 0.505. Meanwhile, *per capita* disposable income, relationship networks, and mental health counseling also negatively influenced residents’ mental health. If *per capita* disposable income is increased by 1 unit, their mental health severity would decrease by 0.206; if the relationship network is increased by 1 unit, their mental health severity would decrease by 0.208. Furthermore, if residents received mental health counseling from the government, the severity of their mental health would be reduced by 0.031. Additionally, some other factors could exacerbate residents’ mental health seriousness. If the proportion of the older adults and children and the time to pay attention to COVID-19 increases by 1 unit, the severity of the mental health of the residents would increase by 0.291 and 0.038, respectively.

### 4.2. Robustness test

#### 4.2.1. Parallel trend test

**Figure 1:**
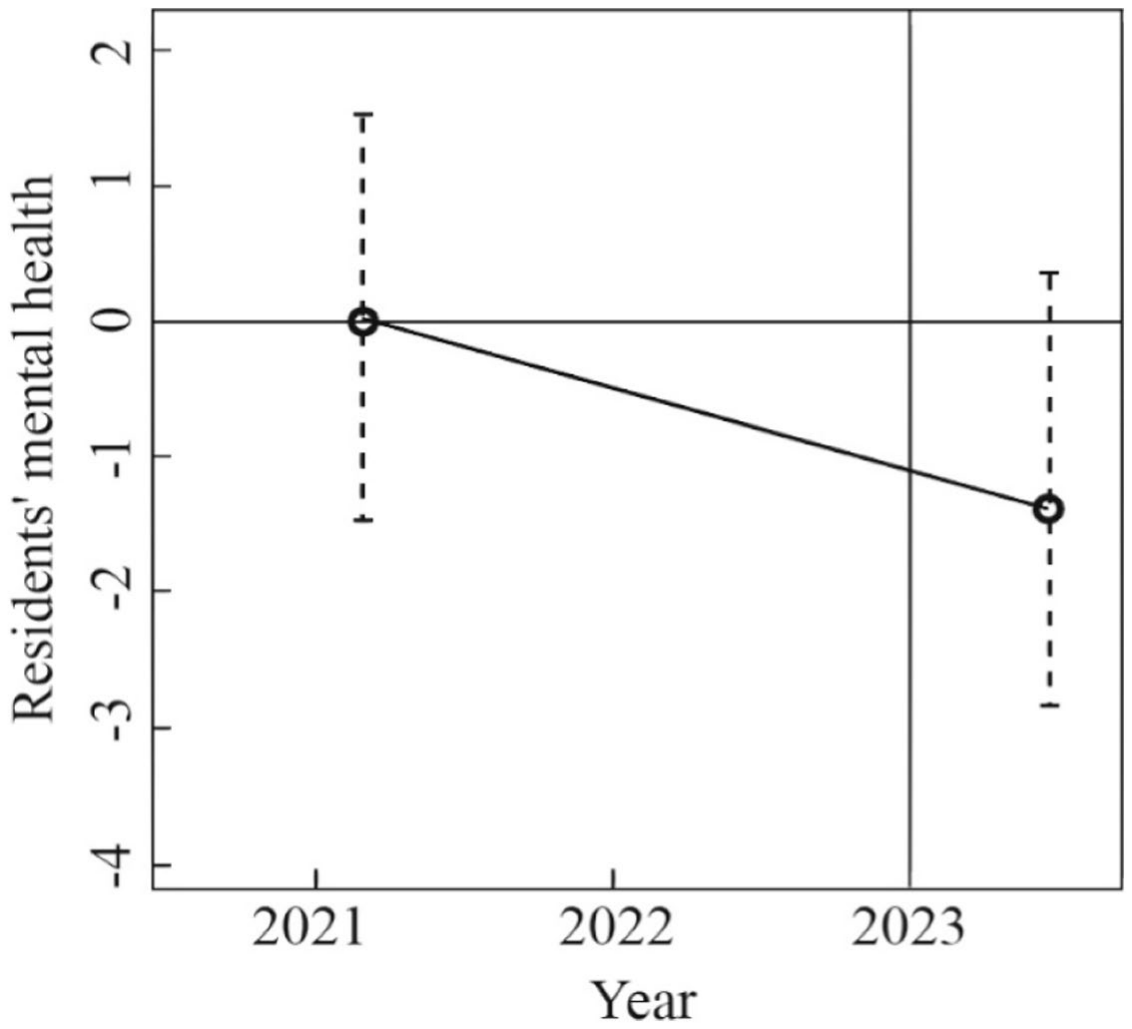
Test results of equilibrium trend.

The DID method required that there was no significant difference in residents’ mental health before December 31, 2022, between the treatment group (samples after the RCC) and the control group (samples before the RCC). According to Figure 1, it is found that there is no significant difference in residents’ mental health before December 31, 2022, while the severity of residents’ mental health decreased significantly during January–March 2023, which further verifies the positive promotion effect of the RCC on improving residents’ mental health.

#### 4.2.2. Placebo test

**Figure 2:**
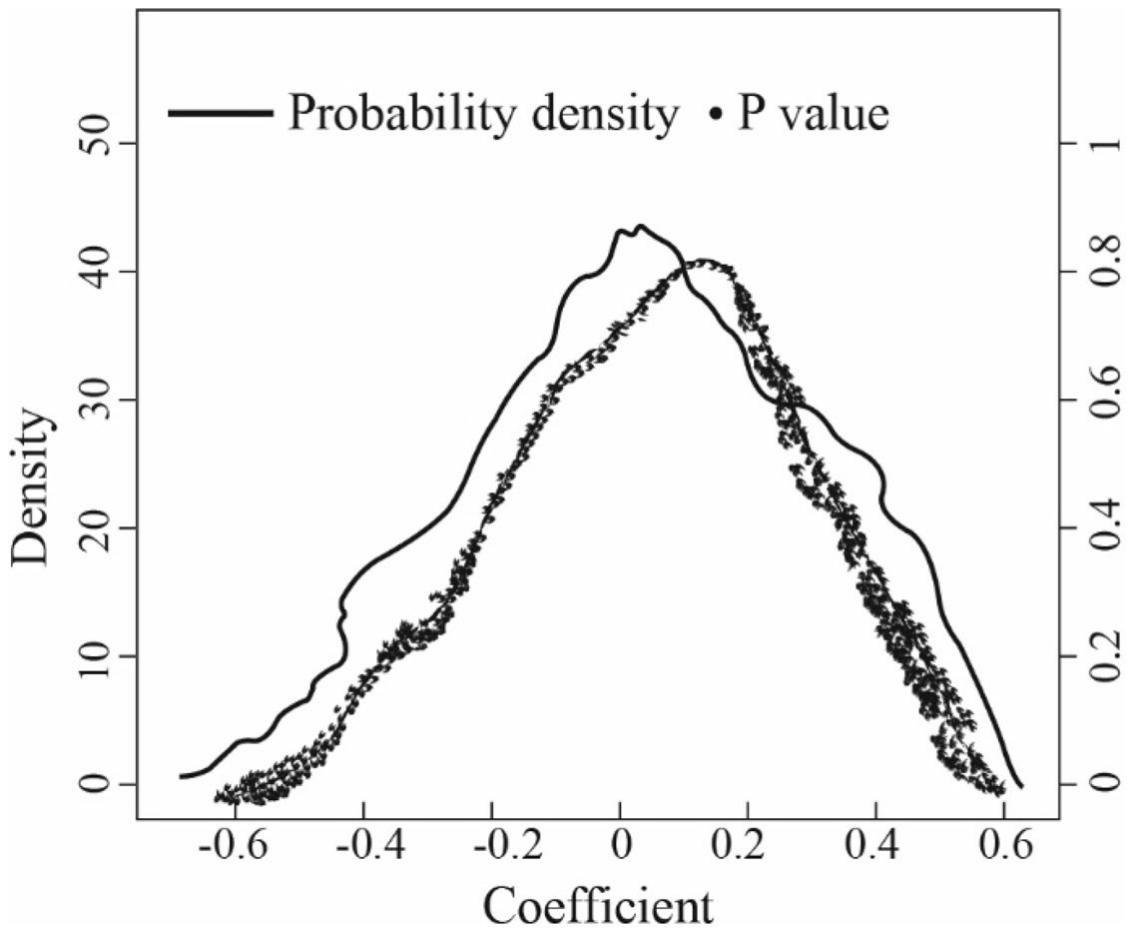
Placebo test result.

Further, exploring whether the estimation results of the DID method are biased or not due to missing variables, this paper conducted a placebo test through a randomized selection of treatment groups. Since the newly generated treatment group was random and would not affect the explained variable, its estimated coefficient should be around 0. This paper repeated the random generation process 1,000 times and reported the estimated coefficient of the random treatment group and its *p*-value distribution in Figure 2. The results showed that the average estimation coefficient of the randomly generated treatment group is-0.0003, which is near 0 and far away from-0.602 in Table 3, indicating that there is no obvious model estimation bias issue.

### 4.3. Heterogeneous effects based on the age of residents and regional differences

Furthermore, Tables 4, 5 showed the influence of the RCC on the mental health of residents of different ages and regions, respectively. Specifically, on the one hand, the RCC did not significantly influence the mental health of residents over the age of 60; that is, the mental health of residents over the age of 60 has not improved considerably after the RCC. However, the RCC showed a linear significant effect on the mental health of residents under 60 years; that is, with the gradual increase of age, the RCC holds a stronger inhibitory effect on the severity of the mental health of residents. Thus, hypothesis H1a is confirmed. On the other hand, the RCC also showed significant effects on the mental health of residents belonging to urban and rural areas. Still, its improvement effect on the severity of the mental health of urban residents is greater than that of rural residents. Thus, hypothesis H1 b is also confirmed (Table 6).

**Table 4:** Heterogeneity analysis based on residents’ age.

Variables	Residents’ mental health			
	Under 20 years old	20–40 years	40–60 years	Over 60 years old
RCC	−0.105** (0.050)	−0.146** (0.066)	−0.164* (0.091)	−0.075 (0.042)
Control variables	Yes	Yes	Yes	Yes
Constant term	0.102*** (0.032)	0.134*** (0.039)	0.099*** (0.027)	0.116*** (0.031)
Fixed area (Urban or rural areas)	Yes	Yes	Yes	Yes
Fixed time (2021.04 or 2023.04)	Yes	Yes	Yes	Yes
R^2^	0.403	0.321	0.405	0.329
Sample size	121	215	262	137

*, **, and *** represent significance at the 10, 5, and 1% levels, respectively. Robust standard error is reported in parentheses.

**Table 5:** Heterogeneity analysis based on regional differences.

Variables	Mental health of residents	
	Urban residents	Rural residents
RCC	−0.372** (0.161)	−0.207* (0.115)
Control variables	Yes	Yes
Constant term	0.165*** (0.046)	0.149*** (0.042)
Fixed time (2021.04 or 2023.04)	Yes	Yes
R^2^	0.421	0.393
Sample size	421	314

*, **, and *** represent significance at the 10, 5, and 1% levels, respectively. Robust standard error is reported in parentheses.

**Table 6:** Test results of the mediating effect of tourism consumption.

Variables	Residents’ mental health	Tourism consumption	Residents’ mental health
RCC	−0.607** (0.282)	0.785* (0.880)	−0.382** (0.173)
Tourism consumption			−0.190*** (0.052)
Control variables	Yes	Yes	Yes
R^2^	0.509	0.421	0.475

*, **, and *** represent significance at the 10, 5, and 1% levels, respectively. The robust standard error is reported in parentheses.

### 4.4. Testing the mediating effect of tourism consumption

This study also used the mediating effect model to verify the mediating mechanism of tourism consumption related to the RCC that influences the mental health of residents. The study selected “amount of family tourism consumption in the first quarter (0–2000 = 1, 2000–4,000 = 2,4,000–6,000 = 3, 6,000–8,000 = 4, more over 8,000 yuan = 5)” to measure mediating variable “tourism consumption.” Using the hierarchical regression model, the results showed that the RCC positively affects tourism consumption. Meanwhile, the RCC and tourism consumption significantly influenced residents’ mental health. The intermediary effect of tourism consumption is 0.1492 (−0.785*0.190), and its proportion in the total effect is 0.2458 (0.1492/0.607). Therefore, 24.58% of the inhibitory effect of the RCC on residents’ mental health severity is found to be contributed by family tourism consumption. Thus, hypothesis H2 is also endorsed.

## 5. Discussion

In infectious disease prevention and control, humanity has experienced the longest, largest, and most damaging COVID-19 outbreak (88, 89). Against climate change, financial turmoil, food crisis, and public health crisis, the COVID-19 pandemic has exacerbated negative impacts on industrial development, international trade, labor employment, and mental health (90–92). This paper focuses mainly on answering the previous discussion in academia; that is, since the COVID-19 epidemic has caused severe damage to residents’ mental health, will residents’ mental health improve after relaxing the control policy of the COVID-19 epidemic? Meanwhile, we discuss the possible mechanism or reason from the perspective of family tourism consumption, unlike many previous studies that only studied the influence of COVID-19 on one aspect, such as the enterprise supply chain, food import and export, household production decisions, and the mental health of residents (10, 93, 94). However, the current study explores the link between RCC, family tourism consumption, and the mental health of residents, which could provide a more plausible explanation for the knock-on effects of COVID-19.

Unlike the study of Wilding et al. (95), Razali et al. (63), and Hecker et al. (96) studies, which focused on the direct causal relationship between COVID-19 and residents’ mental health, our research innovatively and empirically confirms the promotion role of the RCC in improving residents’ mental health. Firstly, the RCC has canceled the home restriction and isolation control policy (97, 98), so that residents can have free activities and seek the best comfortable environment. Second, the RCC accelerates the resumption of business and production, provides adequate employment opportunities (99, 100), and eases the pressure and anxiety of increased family life security and income. Third, the protective antibodies produced by the vaccine and the in-depth understanding of the novel coronavirus have made residents less fearful, and reduced their worries and depression about infection and fear of death (14). Finally, the RCC significantly improves the residents’ face-to-face communication, enhances the strength of the relationship network, and relieves residents’ anxiety and depression (101, 102).

In addition, considering the differences in the ages and regions of the residents, we further analyzed the heterogeneity of the impact of the RCC on the mental health of residents. Although many previous studies have confirmed a causal relationship between the COVID-19 pandemic and mental health in the older adults people (102–104), our study reported insignificant improvement in the severity of mental health in residents over 60 years old after the COVID-19 pandemic was relaxed. Old-age residents are vulnerable to COVID-19, accounting for a high proportion of severe cases and deaths (105). Meanwhile, they also suffer from underlying diseases such as diabetes, asthma, heart or brain infarction, etc. (106). Therefore, given the alternate peaks of COVID-19, the previous mental states of stress, anxiety, and depression are not changed. Moreover, our study confirmed the negative linear inhibitory effect of the RCC on the mental health severity of residents. This is mainly due to easing employment pressure and increasing household income after the COVID-19 control was eliminated (107). Besides, we also find that the RCC improved the mental health of urban residents more than rural residents. Rural residents have apparent advantages over urban residents in terms of the impact of COVID-19 on labor transfer, living security, and epidemic prevention pressure. Thus, they have lower levels of mental health severity and are less affected by changes in COVID-19 control policies (108).

We also explored the impact of other factors on the mental health of residents. Consistent with the studies of Xiong et al. (25) and Wall and Dempsey (109), our study also confirmed that women’s mental health is more serious than men’s; the RCC has a stronger effect on the mental health of male residents. Awareness of COVID-19 helps improve residents’ behavioral decisions about vaccination and production investment (110) but also affects residents’ mental health (111, 112). Our study also showed that the higher the awareness of COVID-19, the lower the severity of stress and anxiety. Furthermore, our findings are supported by Abd-Ellatif et al. (113) and Cheng and Kao (114), who hold that COVID-19 has changed job opportunities, employment environment & income, and decreased residents’ job satisfaction significantly. Furthermore, such negative satisfaction directly aggravates residents’ mental health severity. In addition, it is also found that other factors also exacerbate the seriousness of the mental health of residents. The older adults and children are vulnerable to COVID-19 because they have weak immunity (66, 115). If the older adults and children account for a relatively high proportion of the family population, the stronger the residents’ attention to their health, the more serious the negative emotions of stress and anxiety (63, 116). Of course, residents’ mental health issues are also closely related to their daily life habits. For example, the longer they pay attention to COVID-19, the more they may become too sensitive to the novel coronavirus and self-protection, eventually aggravating their mental health.

Finally, consistent with the studies of Gilbert and Abdullah (117), Chen et al. (118), and Dillette et al. (119), we also find the mediating effect of tourism consumption on improving residents’ mental health influenced by RCC. Firstly, the RCC has changed travel restrictions or quarantine policies due to COVID-19, providing the necessary conditions for the recovery of the tourism industry (120). Secondly, tourism consumption improves residents’ sense of experience and happiness, which can significantly improve the stress, anxiety, and depression caused by COVID-19 (121, 122). Thirdly, tourism consumption improves residents’ social interaction and relationship networks, opens channels for residents to express their emotions and objects to express their emotions, and can reduce mental health problems caused by COVID-19 (49, 123). Finally, studies also confirmed that tourism consumption could repair the long-term impact of COVID-19 on residents’ mental health by adjusting their lifestyles, improving their environment, and communicating with nature (124, 125).

Of course, our research still has some shortcomings. For example, the RCC data was only available for 3 months, and the study only explored the short-term effects of the RCC on the mental health of the residents. Therefore, the long-term effect of the RCC on residents’ mental health needs further investigation. Moreover, some unobserved factors may also affect both the RCC and the mental health of residents, thus generating endogenous issues, which need to be further solved by obtaining survey variables and data. Besides, due to using the panel data of two periods (April 2021 and April 2023) for Shaanxi province, China, we do not have enough data points to test whether there was a pre-trend before the RCC lift.

## 6. Conclusion and policy implications

In the post-epidemic era, in addition to tracking the mutation of the novel coronavirus, designing targeted vaccines, and developing effective drugs, the residents’ mental health also deserves the international community’s attention. The RCC will inevitably lead to economic and social development recovery, but the residents’ mental health resilience is very weak. Whether mental health can recover effectively from RCC also needs theoretical and empirical exploration. In this paper, the panel data of Shaanxi, China, were used to analyze the effects of the RCC on residents’ mental health and the intermediary mechanism of tourism consumption, respectively, by employing the DID method and the mediating effect model. The following conclusions are drawn.

Firstly, the RCC significantly inhibited the mental health severity of residents, and the mental health severity decreased by 0.602. In particular, the RCC has a negative and significant influence on residents’ stress, anxiety, and depression, and the severity of stress, anxiety, and depression decreases by 0.805,0.406, and 0.315, respectively. Hence, the RCC has the strongest negative effect on residents’ stress, followed by anxiety and depression. Secondly, the gender of the residents, awareness of COVID-19, job satisfaction, *per capita* disposable income, relationship network, and mental health counseling also significantly and negatively influence the seriousness of mental health. However, the proportion of older adults and children and the time to pay attention to COVID-19 can exacerbate the severity of mental health. Thirdly, the RCC’s effects on residents’ mental health are heterogeneous. The RCC had a linear significant effect on the mental health of residents under 60 years, while it does not significantly influence the mental health of residents over 60 years. Meanwhile, the RCC’s improvement effect on the mental health severity of urban residents is greater than that of rural residents. Finally, tourism consumption plays a mediating role in the RCC’s influence on residents’ mental health. The intermediary effect of tourism consumption is 0.1492 (−0.785*0.190), and its proportion in the total effect is 0.2458 (0.1492/0.607).

In the post-epidemic era, it is necessary to strengthen mental health interventions. Firstly, the government should strengthen residents’ mental health identification and treatment. Community and public hospitals should increase the number of mental health clinics, expand the scope of identifying residents’ mental health issues, and initiate early intervention strategies. Meanwhile, the government should also improve access to mental health counseling through online consultation platforms, provide mental health relief and self-help information to residents, and help them improve their stress, anxiety, and depression. Secondly, the government should guide enterprises to increase tourism investment, develop tourism products, upgrade tourism formats through financial incentives, and encourage residents to expand tourism consumption to alleviate residents’ mental health issues caused by COVID-19. Thirdly, the government should stimulate economic development, expand the employment of residents, increase household income, and relieve the pressure and anxiety caused by family economic pressure. Finally, the government should continue to track the mutation of the novel coronavirus, forecast timely & provide early warning of new outbreaks, and strengthen the research and development of vaccines and drugs to reduce residents’ fear and concern about COVID-19. Besides, the government should treat residents’ mental health problems from the public health perspective, improve residents’ awareness of mental health, and eliminate social panic & discrimination against mental health.

## Data availability statement

The raw data supporting the conclusions of this article will be made available by the authors, without undue reservation.

## Author contributions

YH: conceptualization & methodology and writing original draft. HS and YD: data sources & review, revise and editing. SZ: funding. All authors contributed to the article and approved the submitted version.

## Funding

This work was supported by the National Social Science Foundation of China (Grant No. 21BKS171).

## Conflict of interest

The authors declare that the research was conducted in the absence of any commercial or financial relationships that could be construed as a potential conflict of interest.

## Publisher’s note

All claims expressed in this article are solely those of the authors and do not necessarily represent those of their affiliated organizations, or those of the publisher, the editors and the reviewers. Any product that may be evaluated in this article, or claim that may be made by its manufacturer, is not guaranteed or endorsed by the publisher.
